# Preventative Effect of Mebendazole against Malignancies in Neurofibromatosis 1

**DOI:** 10.3390/genes11070762

**Published:** 2020-07-08

**Authors:** Verena Staedtke, Tyler Gray-Bethke, Gregory J. Riggins, Ren-Yuan Bai

**Affiliations:** 1 Department of Neurology, Johns Hopkins University School of Medicine, Baltimore, MD 21231, USA; tgraybe1@jhmi.edu; 2 Department of Neurosurgery, Johns Hopkins University School of Medicine, Baltimore, MD 21231, USA; griggin1@jhmi.edu

**Keywords:** neurofibromatosis 1 (NF1), mebendazole (MBZ), COX-2 inhibitor, MPNST, malignancy, sarcoma, chemoprevention

## Abstract

Patients with RASopathy Neurofibromatosis 1 (NF1) are at a markedly increased risk of the development of benign and malignant tumors. Malignant tumors are often recalcitrant to treatments and associated with poor survival; however, no chemopreventative strategies currently exist. We thus evaluated the effect of mebendazole, alone or in combination with cyclooxygenase-2 (COX-2) inhibitors, on the prevention of NF1-related malignancies in a *cis* *Nf1+/−;Tp53+/−* (NPcis) mouse model of NF1. Our in vitro findings showed that mebendazole (MBZ) inhibits the growth of NF1-related malignant peripheral nerve sheath tumors (MPNSTs) through a reduction in activated guanosine triphosphate (GTP)-bound Ras. The daily MBZ treatment of NPcis mice dosed at 195 mg/kg daily, initiated 60 days after birth, substantially delayed the formation of solid malignancies and increased median survival (*p* < 0.0001). Compared to placebo-treated mice, phosphorylated extracellular signal-regulated kinase (pERK) levels were decreased in the malignancies of MBZ-treated mice. The combination of MBZ with COX-2 inhibitor celecoxib (CXB) further enhanced the chemopreventative effect in female mice beyond each drug alone. These findings demonstrate the feasibility of a prevention strategy for malignancy development in high-risk NF1 individuals.

## 1. Introduction

RASopathy Neurofibromatosis 1 (NF1) is an autosomal dominant hereditary cancer predisposition syndrome that affects ~1:3000 individuals [1]. It is caused by mutations in the *neurofibromin 1* (*Nf1*) tumor suppressor gene, which encodes the GTPase-activating protein-related domain (GRD) that catalyzes the inactivation of Ras by accelerating guanosine triphosphate (GTP) hydrolysis to guanosine diphosphate (GDP) [2]. In NF1 individuals, loss of *Nf1* results in high levels of activated Ras, leading to the formation of multiple benign and malignant tumors via multiple effector pathways, including the Ras–MAPK pathway, with subsequent activation of the RAF–MEK–ERK cascade. 

Patients with NF1 have an increased cancer risk and mortality, and lower survival compared with the general population [3,4]. Based on the Finnish NF1 Registry, the estimated lifetime cancer risk in patients with NF1 is 59.6%, with an estimated cumulative cancer risk of ~25% and ~39% by age 30 and 50 years, whereas the respective percentages in the general Finnish population are much lower, at 30.8%, 0.8% and 3.9% [3]. The most common malignancies are of nervous system origin, such as malignant peripheral nerve sheath tumors (MPNSTs) and astrocytomas, which comprise 63% of all malignancies [3]. Other malignancies include breast cancer, rhabdomyosarcomas, pheochromocytoma, gastrointestinal stromal tumor (GIST), malignant fibrous histiocytoma, and thyroid cancer [3].

MPNST is a very aggressive spindle cell sarcoma which accounts for the majority of cancer deaths in all NF1 patients and is a hallmark complication of this condition [3,4,5,6]. MPNST may arise from any of the pre-existing plexiform neurofibromas distributed throughout a patient′s body. Unfortunately, there is no way of knowing which individual and, more specifically, which lesions within any one individual are likely to behave in a malignant fashion and thus many patients require regular screening with standard radiographic techniques such as MRI and PET/CT. Patients with *Nf1* microdeletion, i.e., a large deletion of the *Nf1* gene and its flanking regions, are especially susceptible to MPNSTs [7,8]. 

NF1-specific malignancies, including MPNSTs, typically manifest early in life and are responsible for the relative excess in cancer incidence and mortality observed in children and young adults [4]. Those malignancies are typically very difficult to treat and current therapies have shown little long-term benefit despite extensive research efforts [9]; however, early chemoprevention to delay cancer occurrence and reduce cancer risk remains largely unexplored. The success of chemoprevention has been impressively demonstrated in epithelial malignancies, particularly breast, prostate and colorectal cancers, with the use of selective estrogen receptor modulators (SERM) (e.g., tamoxifen), 5α-reductase inhibitors (e.g., finasteride) and cyclooxygenase-2 (COX-2) inhibitors, a type of non-steroidal anti-inflammatory drug (NSAID, e.g., sulindac, aspirin, celecoxib) that inhibited the appearance of colorectal polyps in various familial colorectal cancer predisposing syndromes [10].

The development of new chemical agents for chemoprevention is a long, difficult and expensive process. A potential strategy to circumvent these challenges is to discover new uses for compounds with an established track record of safe and long-term use in humans, alone or in combination with already known cancer prevention agents, such as widely used cyclooxygenase-2 (COX-2) inhibitors, whose anti-neoplastic effects are mediated through the inhibition of angiogenesis via decreasing COX-2-induced vascular endothelial growth factor (VEGF) production [11] and apoptosis via altered caspase signaling [12,13]. Notably, COX-2 overexpression has been found in a variety of sarcomas and has been associated with poor prognosis [14,15,16], thus suggesting that COX-2 inhibitors could play a role in NF1 cancer prevention. 

We previously identified that mebendazole (MBZ), an FDA-approved low molecular weight benzimidazole derivative with a lengthy track record of safe long-term human use, significantly reduced tumor growth and improved survival in the animal models of glioblastoma multiforme (GBM) and medulloblastoma (Sonic Hedgehog (SHH) Group and c-Myc/OTX2 amplified Group 3) and also reduced tumor formation in a Familial Adenomatous Polyposis (FAP) colon cancer model [17,18,19,20]. A number of mechanisms for MBZ’s anti-neoplastic activity have been proposed by us and others, including microtubule disruption, pro-apoptosis, and the inhibition of growth factor signaling through the blockage of various tyrosine kinases, particularly VEGFR2 [17,18]. 

The current study evaluates the feasibility of a cancer prevention strategy using non-toxic MBZ alone and in combination with COX-2 inhibitors in a *cis Nf1+/−;Tp53+/−* (NPcis) mouse model of NF1 [21]. Like NF1 patients, NPcis mice spontaneously develop predominantly soft tissue sarcomas including MPNSTs (genetically engineered murine (GEM) PNSTs) and malignant Triton tumors, as well as rhabdomyosarcomas and astrocytomas that severely limit their life expectancy to ~5 months [21,22,23,24]. The addition of heterozygous *Tp53* knock-out (KO) accelerates the cancer development, which mimics the secondary mutations required for the transformation to malignancies such as MPNST, where the second copy of *Nf1* is also lost due to the loss of heterozygosity (LOH) [21,22].

## 2. Material and Methods

### 2.1. Tissue Culture and Cell Lines

The human NF1-associated MPNST cell line NF90.8 was provided by Dr. Michael Tainsky (Wayne University, Detroit, MI) and sNF96.2 was purchased from the American Type Culture Collection (ATCC; Manassas, VA, USA). Cells were cultured in DMEM (ATCC) supplemented with 10% fetal bovine serum (FBS) (Sigma, St. Louis, MO, USA) and penicillin/streptomycin (Thermo Fisher, Waltham, MA, USA). These cell lines were not authenticated. All cells were tested and found free of mycoplasma contamination. 

### 2.2. Reagents and Antibodies

Rabbit anti-Nf1 antibody (A300-140A, Lot 3) was purchased from Bethyl Laboratories and anti-βActin horseradish peroxidase (HRP) antibody (C-11, SC-1615HRP, Lot G3015) was purchased from Santa Cruz Biotech. An Active Ras Detection Kit (#8821, antibody Lot 7), including the anti-Ras antibody, was purchased from Cell Signaling Technology. 

### 2.3. Assays

A Ras activity assay was performed according to the manufacturer′s instructions for the Active Ras Detection Kit (Cell Signaling Technology, Danvers, MA, USA). Briefly, cells were lysed with the Lysis/Binding/Wash buffer and pelleted, then the supernatant was used as the cell lysate. In the positive control, 5 µL of 10 mM GTPγS was added to 500 µL of lysates and incubated at 30 °C for 15 min. Cell lysates were incubated with glutathione resin, together with the purified GST-Raf1-RBD protein at 4 °C for 1 h in a spin cup. The resin was washed and the bound proteins were eluted by incubating with dithiothreitol (DTT)-containing sample buffer at RT for 2 min. Eluted samples were heated and analyzed by anti-Ras Western blotting.

A cell proliferation assay was performed using Cell Counting Kit-8 from Dojindo Molecular Technologies. Cells in 100 µL media in a 96-well plate were incubated with 10 µL of WST-8, a tetrazolium salt, at 37 °C in a tissue culture incubator. Absorbance was measured at 450 nm in a PerkinElmer Victor^3^ plate reader. Half maximal inhibitory concentrations (IC_50s_) were determined by incubating cells at a range of concentrations for 72 h and were calculated by GraphPad Prism 5.0 using the log (inhibitor) vs. response function and non-linear fit.

### 2.4. Chemoprevention in NPcis Mice 

NPcis (*cis Nf1+/−;Tp53+/−*) mice in C57BL/6 background (B6;129S2-Trp53tm1Tyj Nf1tm1Tyj/J, Stock No: 008191, Jackson Laboratory) were bred by pairing male heterozygous NPcis mice with the female wildtype mice to better generate MPNST animals [21,23]. Since homozygous *Nf1/Tp53* KO mice are embryonically lethal, only heterozygous and wildtype pups were born [21,25]. Mice were genotyped via qPCR by Transnetyx using the following primer pairs: Nf1 wildtype (WT) (5′-GGTATTGAATTGAAGCACCTTTGTTTGG-3′, 5′-CGTTTGGCATCATCATTATGCTTACA-3′, reporter: 5′-AATATATGACCCCATGGCTGTC-3′), Nf1 KO (5′-TGGAGAGGCTTTTTGCTTCCT-3′, 5′-CGTTTGGCATCATCATTATGCTTACA-3′, reporter: 5′-CTGCTCGACATGGCTG-3′), Tp53 WT (5′-GTGAGGTAGGGAGCGACTTC-3′, 5′-TTGTAGTGGATGGTGGTATACTCAGA-3′, Reporter: 5′-CCTGGATCCTGTGTCTTC-3′) and Tp53 KO (5′-TGTTTTGCCAAGTTCTAATTCCATCAGA-3′, 5′-TTGTAGTGGATGGTGGTATACTCAGA-3′, reporter: 5′-ACAGGATCCTCTAGAGTCAG-3′). At day 60 after birth, heterozygous mice were started on the medicated feed or water. The mouse diet consisting of 45 kcal% fat containing soybean oil and lard for fat (D12451, Research Diets) was used as the control feed. Diets with 175, 195, 215 or 250 mg/kg of MBZ polymorph C (Aurochem Laboratories Ltd., Mumbai, India) or 1000 ppm (mg/kg) celecoxib (Sigma) were manufactured with the D12451 formulation in color codes. Sulindac (Sigma) was added to drinking water at 160 ppm (0.5 mg/day) in 4 mM sodium phosphate buffer as previously described [20]. Animals were palpated weekly for tumors and survival and cause of death, as detailed in the Results section, were recorded. All animal experiments were performed under an approved protocol and in accordance with Johns Hopkins Animal Care and Use guidelines.

### 2.5. Immunohistochemistry

Mouse tumors were first fixed by formalin and embedded in paraffin. For hematoxylin & eosin (H&E) staining, the section was de-paraffinized and stained by the standard hematoxylin and eosin procedure to visualize tissue structures. For immunostaining, rabbit anti-Erk1/2 (Cell Signaling, Cat. No. 9102) and anti-pErk1/2 (Thermo Fisher, Waltham, MA, USA, Cat. No. 36-8800) antibodies were used. Sections were de-paraffinized using a standard procedure and blocked using 1.9% H_2_O_2_ in methanol at room temperature for 10 min. Sections were heated at 100 °C for 20 min in the antigen retrieval citra solution (BioGenex, San Ramon, CA) and blocked by the serum-free protein blocker (Dako, Glostrup, Denmark, Cat. No. X0909) for 5 min at room temperature. After incubation with the rabbit anti-Erk1/2 or anti-pErk1/2 antibody diluted at 1:50 overnight at 4 °C, biotin-conjugated anti-rabbit IgG (Jackson ImmunoResearch, West Grove, PA, USA, Cat. No. 111-066-144) was applied for 20 min at room temperature, followed by washing and incubation with streptavidin peroxidase (Biogenex, Fremont, CA, USA, Cat. No. HK330-9KT) for 15 min at room temperature. Antibody binding was visualized by the 3,3′-Diaminobenzidine (DAB) chromogen system (Dako, Glostrup, Denmark). Subsequently, sections were counterstained by hematoxylin. Immunohistochemistry (IHC) quantification of representative tumor tissue sections was carried out with open source software Fiji ImageJ (NIH, Bethesda, MD, USA) using JPEG files. Mean optical density (OD) was calculated as the log average (maximal intensity/mean intensity) after image processing with color deconvolution and background subtraction.

### 2.6. Statistical Analysis

The results are presented as a mean value plus or minus the standard deviation. Data were analyzed by GraphPad Prism 5.0. The *p*-values were determined by a Mantel–Cox test. A *p*-value under 0.05 was accepted as statistically significant. 

## 3. Results

### 3.1. MBZ Inhibited NF1-Derived MPNST Cell Lines through Ras Inhibition

**Figure 1:**
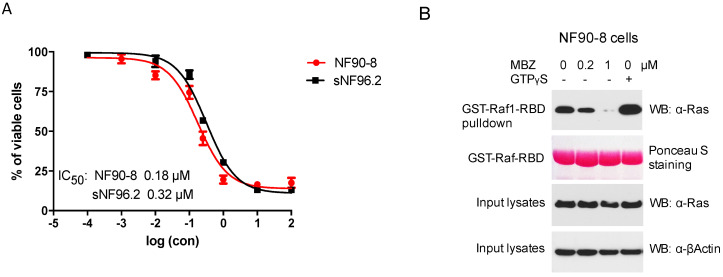
Mebendazole (MBZ) inhibits malignant peripheral nerve sheath tumor (MPNST) cells and Ras activity. (**A**) IC_50s_ of MBZ with NF90-8 and sNF96.2 cells were measured at 0.18 and 0.32 μM, respectively. Cells were incubated with MBZ or DMSO for 72 h and viable cells were determined with WST-8 and calculated as percentage of the control. Data are presented as mean ± s.d. (**B**) RASopathy Neurofibromatosis 1 (NF1)-deficient NF90-8 cells were treated with MBZ at 0.2 and 1 μM for 24 h and cell lysates were incubated with GST-Raf1-RBD (the Ras-binding domain) coupled with glutathione resin. The pulldown products were analyzed by anti-Ras western blot, showing the activated GTP-bound Ras protein. Lysates incubated with GTPγS were used as positive controls.

Human MPNST cells NF90-8 and sNF96.2, both derived from NF1 patients, were treated with MBZ for 72 h at indicated concentrations, revealing favorable IC_50_ levels at 0.18 and 0.32 μM, respectively (Figure 1A). Because NF1-associated tumors are mainly driven by Ras hyperactivation, we studied MBZ’s ability to inhibit Ras activity in the NF90-8 cell line by exposing NF90-8 cells to different concentrations of MBZ (0.2 and 1 μM) for 24 h. The activated form of GTP-bound Ras, detected by GST-Raf1-RBD fusion protein binding, was reduced in MBZ-treated NF90-8 cells in a concentration-dependent manner (Figure 1B). This confirmed the Ras inhibitory effect of MBZ in vitro. 

### 3.2. MBZ Delayed Tumor Formation and Improves Survival in NPcis Mice

As reported before, *cis Nf1+/−;Tp53 +/−* (NPcis) mice are naturally predisposed to a number of solid malignancies, which typically form ~3–5 months after birth: 77% will develop soft tissue sarcomas—of which 60–65% are MPNSTs, 20% malignant Triton tumors, 10% rhabdomyosarcomas, 10% leiomyosarcomas and fibrohistiocytomas, 14% lymphomas, 8% carcinomas, and 1% neuroblastomas [21,22,23]; astrocytomas have also been reported [24,26].

**Figure 2:**
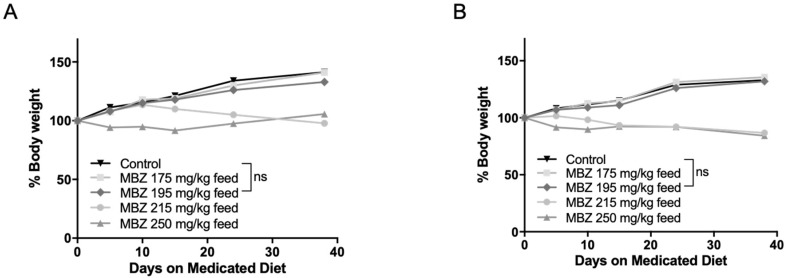
Dose-dependent MBZ toxicity in *cis Nf1+/−;Tp53+/−* (NPcis) mice. The 60-day old NPcis mice were provided with MBZ feed at indicated concentrations. Shown is the 30-day weight of (**A**) male and (**B**) female mice on the MBZ diet with the indicated doses. *n* = 5 mice per each MBZ dosing group.

To determine the most effective and tolerable long-term MBZ dose in vivo, 60-day old male and female NPcis mice were separated into groups and provided with control feed or continuous medicated feed containing 175, 195, 215 or 250 mg/kg MBZ. This range was calculated based on our previously established maximal dose of 50 mg/kg MBZ via oral gavage and the estimated daily food intake of a mouse [17]. Mice were weighed weekly and examined for signs of toxicity over 4 weeks. In the higher MBZ dosing groups of 250 and 215 mg/kg diets, nearly all mice showed evidence of excessive toxicity, including ruffled fur and significant weight loss between 10–15% thereby precluding the long-term use of those doses and establishing 195 mg/kg MBZ feed as the most suitable diet for long-term chemoprevention in these mice (Figure 2A,B). 

In order to investigate the tumor-preventative effects of MBZ, continuous oral administration of MBZ via 195 mg/kg feed was initiated at 60 days after birth, before the formation of any malignancies. Mice were palpated weekly for the presence of any tumors. For the purpose of this study, ‘Solid Malignancies’ were defined as any type of sarcoma and astrocytoma, in addition to neuroblastomas and carcinomas, while ‘Others’ included non-solid malignancies such as lymphomas, leukemias and unknown causes of death. 

**Figure 3:**
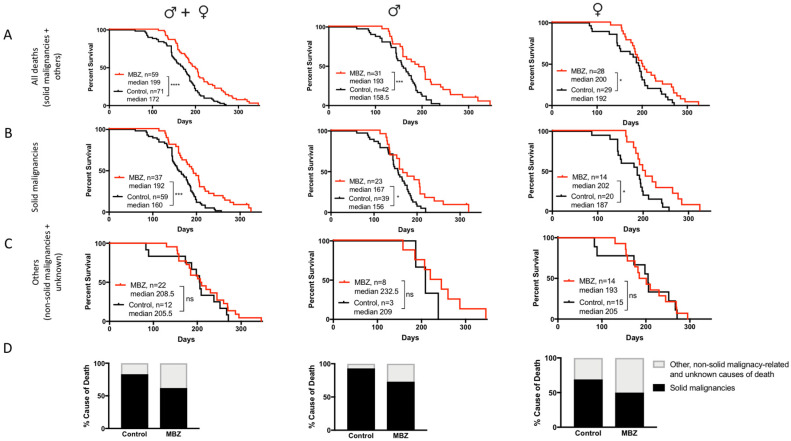
MBZ delays cancer onset and improves survival in NPcis mice. Shown are the Kaplan–Meier curves for MBZ-treated NPcis mice, initiated 60 days after birth, in comparison to controls for (**A**) overall survival, (**B**) solid malignancy-related mortality and (**C**) others, i.e., non-solid malignancy-related and unknown causes of mortality, analyzed as combined (males and females, left) male (middle) and female (right) cohorts. Animal numbers are provided for the specific groups in each graph and were analyzed with a two-sided log-rank test. * *p* ≤ 0.05; *** *p* ≤ 0.001, **** *p* ≤ 0.0001; ns = not significant. (**D**) Percentage distribution of malignancy-related cause of death of MBZ-treated NPcis mice compared to controls, analyzed as combined (males and females, left) male (middle) and female (right) cohorts.

MBZ treatment started at the age of 60 days significantly increased the overall median survival for male, female and combined cohorts (Figure 3A). In MBZ-treated mice, the time to tumor occurrence was significantly delayed compared to untreated control animals: 50% of all control mice had developed tumors and succumbed to disease by the age of 160 days, whereas in the MBZ-treated cohort, the tumor occurrence and median mortality was delayed by 32 days to 192 days (Figure 3B). Although observed in male and female NPcis mice alike, MBZ′s cancer preventative effect appeared to be more pronounced in males, with an increase in median survival by 34.5 days compared to 14 days in female mice (Figure 3B). Figure 3C demonstrates that MBZ′s chemopreventative effect was specific to mice with solid malignancies and did not affect the median survival of other, i.e., non-solid malignancy-related and unknown, causes of death both in male and female mice (Figure 3C). Lastly, MBZ treatment resulted in a ~25% reduction in solid cancer-related causes of death, thus demonstrating the feasibility of such a cancer prevention strategy in these NPcis mice (Figure 3D)

### 3.3. MBZ Reduced pERK Activity in Tumors In Vivo

**Figure 4:**
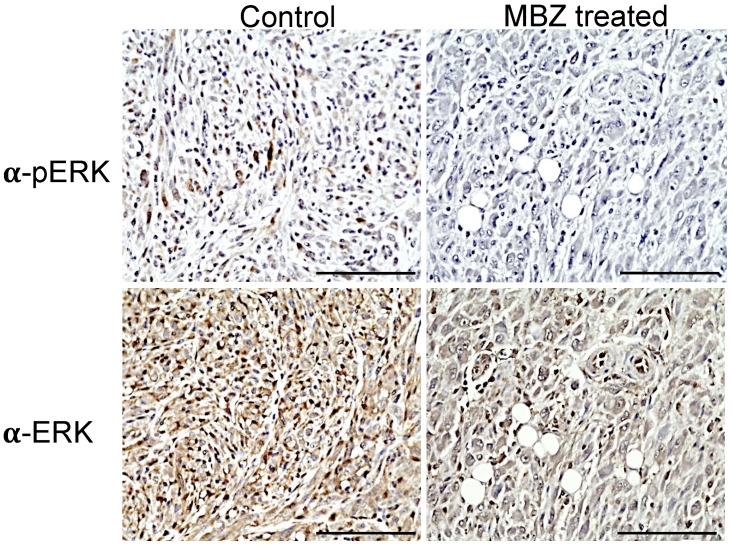
MBZ reduces ERK (pERK) in treated NPcis mice. Representative images of tumors from untreated controls (left) and MBZ-treated NPcis mice (left) were stained for pERK1/2 (upper row) and ERK1/2 (lower row). pERK staining was visualized in brown in untreated controls but reduced in tumors of MBZ-treated mice. Each scale bar represents 100 µm.

In NPcis mice, the loss of *Nf1* leads to the hyperactivation of Ras, with the subsequent activation of the downstream effector ERK that is reflected by elevated levels of phosphorylated ERK (pERK) in MPNSTs and other related tumors. Immunohistochemistry showed that continuous MBZ treatment with a 195 mg/kg diet reduced pERK levels in sarcomas of NPcis mice compared to untreated mice (Figure 4). An analysis of the DAB staining intensity in three independent MBZ-treated tumor samples confirmed these results, with a reduced mean optical density (OD) of 0.02 in MBZ-treated samples compared to 0.05 in controls, while ERK staining was similar between both groups, with mean intensities of 0.07 and 0.08 for MBZ-treated and untreated tumors, respectively (Figure 4). 

### 3.4. Cancer-Preventative Effects of CXB and MBZ Are Similar in NPcis Mice

**Figure 5:**
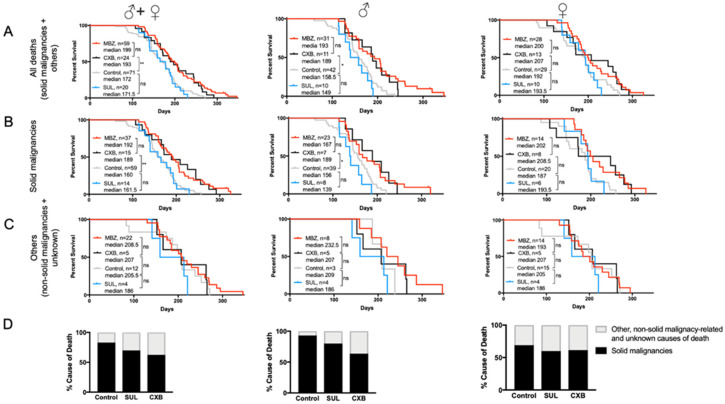
MBZ and cyclooxygenase-2 (COX-2) inhibitor celecoxib (CXB) are similarly effective in preventing cancer in NPcis mice. Shown are the Kaplan–Meier curves for CXB and sulindac (SUL)-treated NPcis mice, initiated 60 days after birth, in comparison to MBZ-treated mice and untreated controls for (**A**) overall survival, (**B**) solid malignancy-related mortality and (**C**) other, non-solid malignancy-related and unknown causes of mortality, analyzed as combined (males and females, left) male (middle) and female (right) cohorts. Animal numbers are provided for the specific groups in each graph and analyzed with two-sided log-rank test. * *p* ≤ 0.05; ** *p* ≤ 0.01; ns = not significant. (**D**) Percentage distribution of solid malignancy-related cause of death by CXB and SUL treated NPcis mice compared to controls, analyzed as combined (males and females, left) male (middle) and female (right) cohorts.

The antitumor effect of selective COX-2 inhibitors, such as sulindac (SUL) and celecoxib (CXB), has been shown in several malignancies and cancer predisposition syndromes. In the NPcis mouse model, we found that MBZ-treated mice had a longer overall median survival of 199 days compared to CXB, with 193 days; however, this difference was not statistically significant (Figure 5A). When compared to untreated controls, CXB′s effect on median survival was statistically increased in male NPcis mice with solid malignancies, while female mice showed a notable, but statistically insignificant, increase in survival compared to controls. Furthermore, CXB was substantially more effective in delaying the onset of malignancies than SUL, which showed a median survival of 171.5 days and failed to demonstrate any effect in male or female mice compared to controls (Figure 5A,B). Like MBZ, neither SUL nor CXB had an effect on the survival of non-cancer related causes (Figure 5C). Consistent with our findings, we also noticed a ~25% decline in cancer-related cause of death in CXB-treated mice (Figure 5D). 

### 3.5. MBZ Is More Effective than Combined MBZ with CXB

**Figure 6:**
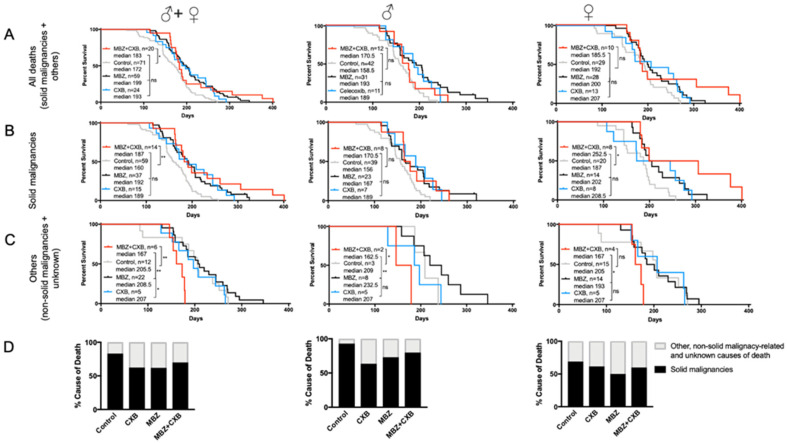
Combination of MBZ and CXB enhances survival in female NPcis mice. Shown are the Kaplan–Meier curves for NPcis mice treated with combined MBZ and CXB, initiated 60 days after birth, in comparison to CXB and MBZ alone for (**A**) overall survival, (**B**) solid malignancy-related mortality and (**C**) other, non-solid malignancy-related and unknown causes of mortality, analyzed as combined (males and females, left) male (middle) and female (right) cohorts. Animal numbers are provided for the specific groups in each graph and analyzed with two-sided log-rank test. * *p* ≤ 0.05; ** *p* ≤ 0.01; ns = not significant. (**D**) Percentage distribution of solid malignancy-related cause of death by combined MBZ/CXB treated NPcis mice compared to MBZ, CXB and controls, analyzed as combined (males and females, left) male (middle) and female (right) cohorts.

Combined treatment with MBZ and CXB significantly increased median survival in NPcis mice compared to controls. However, the observed overall survival benefit appeared inferior to the effect achieved by MBZ or CXB alone, however, the difference is not statistically significant (Figure 6A). When investigating gender-specific effects, we found that dual use of MBZ and CXB in female NPcis mice was successful in delaying solid cancer occurrence and substantially enhancing the median survival beyond what was achieved by each agent alone and untreated controls (Figure 6B). This stands in contrast to male mice with solid malignancies, who did not experience any additional survival benefits from the combination treatment in comparison to single agent MBZ or CXB (Figure 6B). Figure 6C demonstrated that the combination therapy of MBZ and CXB resulted in an earlier mortality from non-solid cancer-related causes, particularly for male mice, indicating possibly the presence of toxicity, which we had assessed beforehand for each agent separately but not in combination (Figure 6C). However, the number of mice who died in the MBZ/CXB cohort due to other, non-solid malignancy-related and unknown causes, were small and thus, limiting our ability to conclusively interpret these results. When analyzing cause of death in MBZ/CXB-treated mice, we observed a reduction in solid cancer-related causes in comparison to the controls, as expected, which was largely comparable with what was seen with single agent use (Figure 6D).

## 4. Discussion 

Our previous work showed that MBZ′s anti-tumor effect in glioblastomas and medulloblastomas is caused by multiple different mechanisms, such as the inhibition of microtubule formation and VEGFR2 autophosphorylation [17,18], which was corroborated by other investigators, applied to various preclinical cancer models and ultimately translated into clinical trials for adult and pediatric patients with cancer (NCT03925662, NCT03628079, NCT02644291, NCT01729260, NCT01837862). In the current study, we expanded MBZ’s scope of application to chemoprevention, i.e., the use of drugs to reduce the risk of cancer development, in high-risk patients of NF1. NF1 is the most common tumor predisposition syndrome in which the loss of tumor suppressor neurofibromin leads to the activation of the Ras proto-oncogene and the development of dozens of benign and malignant tumors. MPNSTs and gliomas are the most common NF1-specific cancers, accounting for 63% of malignancies and a substantial mortality burden in adults younger than 40 years of age; other sarcomas (e.g., rhabdomyosarcomas), gastrointestinal stromal tumors, pheochromocytomas and breast cancers may also occur at a higher frequency compared to the non-affected population [3,4]. MPNSTs in NF1 patients have been particularly recalcitrant to treatment, with overall survival times that are shorter than those of patients with spontaneous MPNSTs. Surgical removal of a high-risk, pre-cancerous lesion is the only prophylactic modality that may reduce mortality, but has unfortunately been associated with morbidity. 

In this study, we found that MBZ inhibited the growth of NF1-related MPNST cells in vitro and substantially delayed tumor formation in NPcis mice when initiated 60 days after birth, without overt disease. Interestingly, the effect was different between genders, with male mice experiencing a more substantial protective effect than female mice, who tend to develop tumors later than their male counterparts and have a longer median survival. A similar observation was made in mice treated with combined CXB with MBZ, which resulted in the largest delay in tumor occurrence and superior median survival in female mice, while males did not experience any benefit from the combination therapy compared to single agent MBZ or CXB. These potential gender-linked distinctions of cancer preventatives are important to realize, as this could impact the clinical applicability of such agents and patient management. Notably, the male bias of NPcis mice in developing MPNST has been reported before [27]. One could also envision intrinsic factors such as the tumor microenvironment, inflammation and differences in the sex hormones as potential causes of this phenomenon [26,28,29]; however, the underlying mechanism is unclear and should be investigated further in animals and humans. Our data further suggest that MBZ′s cytotoxic effect on NF1-related malignancies may result from a reduction in activated GTP-bound Ras and a subsequent decrease in pERK in MBZ-treated malignancies in vivo, thus directly targeting the molecular underpinnings for tumor development in this condition. The potential significant impact of such chemoprevention on the mortality rate of cancer in the NF1 patient population can be envisioned from the success of NSAIDs and other agents on reducing the risk of colorectal, prostate and breast cancer. We therefore hope that, by demonstrating the feasibility of a chemopreventative approach for NF1, this will stimulate a rational approach to interrogate already existing databases for drugs that appear to decrease Ras activity and/or increase NF1 expression as a preventative drug discovery pipeline in these patients in order to reduce cancer occurrence and mortality. 

Chemoprevention may involve the perturbation of a variety of steps in tumor initiation, promotion and progression. As such, COX-2 overexpression leads to cancer cell proliferation, neovascularization, and suppression of apoptosis and thus is associated with a worse prognosis in various malignancies, especially sarcomas [14,15,16]. It is therefore not surprising that overexpression of COX-2 has also been observed in NF1-associated MPNSTs and that selective COX-2 inhibition had an antitumor effect on these cells [30]. Our study confirmed these results and showed that the selective COX-2 inhibitor CXB, but not the non-selective COX inhibitor SUL, delayed cancer occurrence and increased median survival in both male and female NPcis mice. 

Effective chemoprevention requires the need to identify a high-risk patient population and compounds or drug combinations with very low toxicity to allow long-term use in humans. When initiated 60 days after birth, long-term daily continuous MBZ administration was well tolerated in male and female NPcis mice, with stable weights using 195 mg/kg MBZ feed. This is in line with human data, which demonstrate a >40-year history of safe and continuous use for parasitic infections and cystic echinococcosis. This, along with the observed Ras inhibitory effect, could make MBZ an attractive candidate for long-term chemoprevention in the NF1 patient population. It should be noted that rigorous monitoring for adverse reactions would be required, as unexpected and expected toxicities could develop from long-term use of cancer preventative agents, particularly when multiple agents are used, and the benefits should clearly outweigh any potential risks. Given the heterogeneity of clinical symptoms among NF1 patients, it is doubtful that all NF1 patients would experience the same benefits and patient groups at high or low risk would have to be defined. For example, the low-risk NF1 population would include individuals with NF1 Arg1809, NF1 Arg1038Gly, NF1 Met992del, and NF1 Met1149 mutations, all of which are known not to develop any tumors or malignancies [31,32,33,34]. In contrast, the largest benefit would likely be observed in patients with a severe phenotype characterized by a higher tumor burden and a higher risk of malignancies. This group of patients would include individuals with large *Nf1* microdeletions, in which the lifetime risk for MPNST is increased to 16–26% [7,35]; patients with an NF1 p.844–848 missense mutation, who have a higher predisposition for symptomatic neurofibromas, optic pathway gliomas and malignancies compared with the general NF1-affected population [36] and NF1 patients with Arg1276 variants, who are also at a higher risk of developing symptomatic tumors and MPNSTs [31]. 

In summary, this study lays an important foundation for the effective and feasible chemoprevention of malignancies in patients with NF1, which has the potential to delay or perhaps even prevent the malignant transformation of MPNST and other NF1-related malignancies, decrease the need for surgical intervention and reduce the use of antineoplastic therapies in this patient population. Further research is necessary to evaluate these findings in a larger animal studies, such as the NF1 pig model, and to determine whether the observed effects will result in improved clinical outcomes.
